# Circulating Exosomal miRNAs as Biomarkers in Epithelial Ovarian Cancer

**DOI:** 10.3390/biomedicines9101433

**Published:** 2021-10-10

**Authors:** Meng-Shin Shiao, Jia-Ming Chang, Arb-Aroon Lertkhachonsuk, Naparat Rermluk, Natini Jinawath

**Affiliations:** 1 Research Center, Faculty of Medicine Ramathibodi Hospital, Mahidol University, Bangkok 10400, Thailand; msshiao@gmail.com; 2 Department of Computer Science, National Chengchi University, Taipei 11605, Taiwan; chang.jiaming@gmail.com; 3 Division of Gynecologic Oncology, Department of Obstetrics and Gynecology, Faculty of Medicine Ramathibodi Hospital, Mahidol University, Bangkok 10400, Thailand; arbaroon.let@mahidol.ac.th; 4 Department of Pathology, Faculty of Medicine Ramathibodi Hospital, Mahidol University, Bangkok 10400, Thailand; naparat.rer@mahidol.ac.th; 5 Ramathibodi Comprehensive Cancer Center, Faculty of Medicine Ramathibodi Hospital, Mahidol University, Bangkok 10400, Thailand; 6 Integrative Computational Bioscience Center (ICBS), Mahidol University, Nakhon Pathom 73170, Thailand; 7 Program in Translational Medicine, Faculty of Medicine Ramathibodi Hospital, Mahidol University, Bangkok 10400, Thailand

**Keywords:** exosome, miRNA, biomarkers, epithelial ovarian cancer, liquid biopsy

## Abstract

Failure to detect early-stage epithelial ovarian cancer (EOC) is a major contributing factor to its low survival rate. Increasing evidence suggests that different subtypes of EOC may behave as distinct diseases due to their different cells of origins, histology and treatment responses. Therefore, the identification of EOC subtype-specific biomarkers that can early detect the disease should be clinically beneficial. Exosomes are extracellular vesicles secreted by different types of cells and carry biological molecules, which play important roles in cell-cell communication and regulation of various biological processes. Multiple studies have proposed that exosomal miRNAs present in the circulation are good biomarkers for non-invasive early detection of cancer. In this review, the potential use of exosomal miRNAs as early detection biomarkers for EOCs and their accuracy are discussed. We also review the differential expression of circulating exosomal miRNAs and cell-free miRNAs between different biofluid sources, i.e., plasma and serum, and touch on the issue of endogenous reference miRNA selection. Additionally, the current clinical trials using miRNAs for detecting EOCs are summarized. In conclusion, circulating exosomal miRNAs as the non-invasive biomarkers have a high potential for early detection of EOC and its subtypes, and are likely to be clinically important in the future.

## 1. Introduction

Ovarian cancer (OC) is the eight most common cancer in women worldwide and remains the leading cause of mortality among gynecological malignancies in developed countries [1,2]. The estimated numbers of new OC cases and death in 2020 in the United States are 21,750 and 13,940, while globally the numbers are 313,959 and 207,252, respectively [1,2]. OC is mostly asymptomatic in its early stages; thus, the patients usually present with advanced-stage cancer, which is commonly associated with high mortality rate. Therefore, the development of a non-invasive diagnostic approach to accurately detect the disease early is one of the holy grails of combating OC and would lead to the increased overall survival. 

Around 90% of the OC tumors are of epithelial origin (epithelial ovarian cancer; EOC), which can be further subdivided into different histopathological subtypes. Several EOC subtype classification systems have been proposed. The most widely applied system in the clinical practice was proposed by the World Health Organization (the WHO classification system) [3]. According to the recent WHO classification of tumors of the ovary, there are seven major subtypes of EOC: serous carcinoma, mucinous carcinoma, endometrioid carcinoma, clear-cell carcinoma, seromucinous carcinoma, malignant Brenner tumor and undifferentiated carcinoma. These tumors are mainly classified based on their histomorphological features; i.e., serous carcinoma has columnar cells resemble those of tubal-type epithelium; mucinous carcinoma has gastrointestinal-type mucin producing cells; clear-cell carcinoma has cells with clear cytoplasm with characteristic “hobnail” appearance; endometrioid carcinoma has cells resemble to the endometrial glands; Brenner tumors have cells similar to transitional/urothelial epithelium; seromucinous carcinoma has cells with both serous and endocervical-type mucin producing cells; and undifferentiated carcinoma have monotonous non-cohesive cells with lack of defining cell types. Furthermore, the serous carcinoma is classified into high-grade and low-grade serous carcinoma, which is based on the different degree of cytologic atypia and mitotic rate. High-grade serous carcinoma is featured with clear cytoplasm and pleomorphic/bizarre nucleus. Nuclei atypia with more than 12 mitoses per 10 high power fields in the worst area of tumors, multinucleated tumor giant cells and tumor architecture resemble epithelial cells of fallopian tube origin are also often seen in high-grade serous carcinoma [4,5,6]. Another system, dualistic classification of primary EOCs, is also widely applied in many research practices [7,8,9]. This system divides EOCs into two main types, type I and type II, by integrating the histopathologic classification with the molecular genetics findings. Currently, type I tumors, which have a relatively better clinical outcome, are subdivided into three groups: (i) endometriosis-related tumors that include endometrioid, clear cell and seromucinous carcinomas; (ii) low-grade serous carcinomas; and (iii) mucinous carcinomas and malignant Brenner tumors, while type II tumors are composed of the more aggressive high-grade serous carcinoma, carcinosarcoma and undifferentiated carcinoma [10]. 

Accurate diagnosis of the stage and subtype of EOC is very important because standard treatment options affect each subtype differently. The combination of paclitaxel and carboplatin chemotherapy usually produces good initial response rates in high-grade serous carcinoma (60–80%), but eventually most patients become platinum resistant and succumb to subsequent relapses. However, the majority of clear cell, mucinous and low-grade serous carcinoma are resistant to platinum chemotherapy, resulting in reduced usage of platinum for these subtypes. It has now become a standard practice to identify serous vs. non-serous EOC subtypes so that the suitable treatment can be selected for EOC patients [11].

## 2. Current Epithelial Ovarian Cancer (EOC) Biomarkers

In combination with histomorphology, immunohistochemistry (IHC) can help distinguish difficult-to-diagnose EOC subtypes [12,13,14]. p53 IHC is routinely used to distinguish low-grade from high-grade serous carcinoma; the pattern of p53 IHC staining in high-grade serous carcinoma is all or none (overexpression or complete absence), which reflects the underlying TP53 mutation. The combination of WT1 and p53 can be used to distinguish serous carcinoma from endometrioid carcinoma, while the combination of WT1, Napsin A and ER are used to distinguish clear-cell carcinoma from serous carcinoma. ER alone is used to distinguish endometrioid carcinoma from mucinous carcinoma. Recent studies from Kobel et al. proposed the use of the eight IHC biomarkers, namely WT1, p53, p16, Napsin A, PGR, TFF3, ARID1A and VIM, to classify subtypes of a large cohort of EOCs based on nominal logistic regression model [13,14]. After comparing the original diagnosis with the predicted histotypes, the IHC panel could correctly reclassify ~93% of the cases [13,14]. The most common misclassification involved reclassification from high-grade endometrioid to high-grade serous carcinoma, which are the two subtypes known to be difficult to distinguish by histomorphology. Additionally, supported evidence such as molecular alterations and clinical behaviors can help increase the accuracy of the subtype diagnosis.

In terms of molecular alterations, high-grade serous carcinoma is usually associated with *BRCA1* or *BRCA2* germline mutations and *TP53* somatic mutations, which are identified in 96% of the tumor samples [15]. Other than the three genes, mutations in *CSMD3*, *NF1*, *CDK12* and *RB1* are also commonly found [15]. Low-grade serous carcinoma has distinct underlying molecular mechanism than that of high-grade serous carcinoma. It is associated with *KRAS* and *BRAF* mutations but not *TP53* mutations [16,17]. Although it is believed that clear-cell and endometrioid carcinomas share similar molecular genetic profiles as they are both proposed to originate from endometriosis [18], the morphology and clinical behavior of the two subtypes are different. Genetic alterations in *ARID1A*, *PIK3CA* and *PTEN* occur in both subtypes, while microsatellite instability and *CTNNB1* mutations are more commonly observed in endometrioid carcinoma [10,19,20,21]. A recent study by Cochrane et al. proposed that clear-cell and endometrioid carcinomas may originate from different cell types of endometria as clear-cell tumors express much higher level of markers of the ciliated cells (cystathionine gamma-lyase (CTH), etc.), while endometrioid tumors express markers of the secretory cells of the endometrium (methylenetetrahydrofolate dehydrogenase 1 (MTHFD1) and ER) [22]. The authors further posited that clear-cell carcinoma may originate from the progenitor of ciliated cells. For mucinous carcinoma, *KRAS* alterations are found in more than 50% of the tumors [23,24,25,26,27]. 

Clinical screening of EOC includes imaging and serum biomarkers. Imaging tests include transabdominal and transvaginal ultrasound screening (TVS), computed tomography (CT) scan, magnetic resonance imaging (MRI) and positron emission tomography (PET) scan. Biomarker tests in the blood include the commonly used serum tumor markers; CA-125, cancer antigen 19-9 (CA19-9) and carcinoembryonic antigen (CEA). Most of the currently available tests give relatively low sensitivity and specificity, making them unable to detect early-stage EOCs and thus resulting in no significant changes of overall survival of EOC patients in the past 20 years [28]. The recently published study showed that neither the annual screening using serum CA-125 nor TSV can help reduce the mortality rate of ovarian cancer. This large randomized controlled trial in the UK, which recruited 202,562 postmenopausal women who were 50–74 years of age and conducted more than 16 years of follow-up, provides definitive new evidence that the existing general population screening approaches did not reduce ovarian cancer deaths [29]. Currently, OVA1 and its second-generation multivariate index assay (MIA), OVERA, are among the commonly used US Food and Drug Administration (FDA)-cleared assays that assess malignancy risk in adnexal masses planned for surgery. OVA1 has five serum protein biomarkers including CA-125, Transthyretin, Apolipoprotein A1, Transferrin and B2-microglobulin. OVERA substitutes B2-microglobulin and Transthyretin used in OVA1 with Human Epididymis secretory protein 4 (HE4) and Follicle-Stimulating Hormone (FSH). Both assays are used in combination with clinical assessment in women who have a pelvic mass to assess ovarian cancer risk prior to surgical treatment planning and are not a screening test. In addition, although OVA1 and OVERA have high sensitivities (92%, 91%), the specificities are quite low (42%, 69%) [30,31]. Therefore, it is critical to establish new strategies to efficiently screen the disease in its early stage, which is key to improving survival rate.

Taken together, novel biomarkers for early detection of EOCs, which are more effective and able to distinguish the subtypes to facilitate a timely clinical decision are urgently needed. Lately, exosomes have been extensively studied with an increasing number of reports showing their potential as a rich source of biomarkers. Exosomes are a type of extracellular membrane vesicles (EVs) with a diameter of 40–100 nm that are secreted by all cell type. Exosomes carry various biological molecules such as protein, lipid, mRNA and non-coding RNA including microRNA (miRNA) and long non-coding RNA (lncRNA) and transport them to a distant location via the circulatory system. They can be detected in various kinds of body fluids such as blood, urine and saliva. Several pathways have been proposed for biogenesis of exosomes [32]. One of the most well-studied pathways involves the generation of intracellular multivesicular bodies (MVBs) in two steps. First, the invagination of the plasma membrane and the formation of MVBs, which contains intraluminal vesicles (ILVs). Following the formation of MVBs, ILVs will be secreted to the extracellular compartment through the fusion of MVB to the plasma membrane and exocytosis, and they are now called exosomes. Exosomes are continuously being generated by and taken up by cells. Exosomes that are taken up are subsequently degraded by lysosomes or fused with preexisting early-sorting endosomes before disintegrating and releasing their contents into the endoplasmic reticulum and/or cytoplasm. Due to their lipid bilayer encapsulation, enzyme-sensitive molecular cargos are well preserved in exosomes [33]. 

Recent studies showed that exosomes play important roles in cell-cell communication and are particularly enriched in tumor microenvironment [34,35,36]. Among the biomolecules carried by exosomes, miRNAs are the most abundant, and have been shown to facilitate motility and invasiveness of ovarian cancer cells [37,38]. In addition, exosomes secreted by stromal cells may promote drug resistance of cancer cells [34,39,40]. One of the advantages of using miRNAs as liquid biopsy-based biomarker is that they are relatively stable in biofluids, which is particularly important because most of the specimens may not be processed immediately after collection in the clinic. Exosomal miRNAs are better protected from RNase degradation and are therefore more frequently studied as potential biomarkers than the non-exosomal circulating miRNA counterparts [41]. In this review, we focus on all the published exosomal studies so far that showed sensitivity and specificity of exosomal miRNAs in diagnosing and/or predicting the progression of EOC and its subtypes. Moreover, to address which biofluid source is suitable for circulating miRNA biomarker discovery, we summarize the reports showing differential profiles of circulating exosomal and cell-free miRNAs in different blood components (i.e., serum, plasma, platelet). As miRNAs in exosomes are only a small fraction of the entire transcriptomes, the optimal endogenous controls for expression profile normalization are still under debate, and thus we also discuss this topic in detail here. This comprehensive review of the exosomal miRNA biomarkers should provide more information for the future development of early detection biomarkers for EOCs. 

## 3. Exosomal miRNAs as Diagnostic and Prognostic Biomarkers for EOCs

The first study that proposed the use of circulating exosomal miRNAs as diagnostic biomarkers for EOCs was published in 2008 by Taylor and Gercel-Taylor [42]. The authors identified eight exosomal miRNAs specifically up-regulated in the serum of patients with serous papillary adenocarcinoma, which is now referred to as high-grade serous carcinoma (Table 1). These eight miRNAs included the miR-200 family members (miR-200a, -200b, -200c, -141), miR-21, miR-203, miR-205 and miR-214. MiR-200c and miR-214 also showed higher expression in the patients with stage II and III disease comparing to those with stage I. The authors further concluded that since the expression of these miRNAs were similar between the ovarian tumor tissues and the circulating exosomes, miRNA profiling of the circulating exosomes could potentially be used as surrogate diagnostic markers for EOC instead of using a tissue biopsy. 

Meng et al. (2016) examined the possibility of using four circulating exosomal miRNAs in the serum to detect EOCs [43] (Table 1). The four exosomal miRNAs, miR-373 and miR-200 family members (miRNA-200a, 200b and 200c), were selected for studying as they were proposed to be associated with EOCs and breast cancers in the literature [48,49]. The authors observed significant up-regulation of the four exosomal miRNAs in EOCs as compared with benign tumors and healthy controls. However, no differential expression of the 4 miRNAs was observed in different EOC subtypes. Diagnostic performance was further tested by using each miRNA alone or by using a combination of all three miRNAs from the miR-200 family. The AUC values ranged from 0.655 to 0.914 using each miRNA and increased to 0.925 when using a model with all three miR-200 family members (Table 2). In addition, the authors found that the increased expression levels of miR-200b and miR-200c were associated with advanced stages, lymph node metastasis, high CA-125 values and a shorter overall survival, which indicates the potential of using these two miRNAs as prognostic biomarkers for disease progression. Of note, the authors also observed very low expression of the other two members of miR-200 family, i.e., miR-141 and miR-429, in EOC, which contradicts the findings in the study by Taylor and Gercel-Taylor [42]. 

The same team later examined a collective list of 44 miRNAs with oncogenic or tumor suppressive function in EOC from the literature and further tested their quantities in the circulating plasma exosomes [44]. A total of 106 EOC patients, who mostly had high-grade serous carcinoma, and 29 healthy participants were compared. Among the 44 miRNAs, four (miR-21, -100, -200b and -320) were up-regulated, and four (miR-16, -93, -126 and -223) were down-regulated in the EOC cases (Table 1). The diagnostic performance using each of the four up-regulated miRNAs showed miR-200b as having the highest AUC (0.868) (Table 2). The author also pointed out some contradictory findings; although increased miR-200b expression in the circulating exosomes of EOC patients is associated with poorer prognosis [43], its overexpression in ovarian cancer cell lines resulted in reduced proliferation and increased apoptosis [50]. MiR-200b seems to have a dual role in EOC as it has been reported as having both oncogenic and tumor suppressive functions [51]. In addition, miR-200c, previously reported as having a significantly higher expression in the serum of EOC patients than in the healthy subjects [42,43], showed no significant differences between the two groups in this study.

Kobayashi et al. (2018) observed significant up-regulation of exosomal miR-1290 secreted by high-grade serous carcinoma cell lines in comparison to an immortalized normal ovarian epithelial cell line, and thus further examined the circulating cell-free miR-1290 in the serum of EOC patients [45]. A significant up-regulation of miR-1290 was observed in patients with high-grade serous carcinoma comparing to healthy controls (Table 1). The sensitivity and specificity of EOC detection were estimated in different subtypes by using miR-1290 expression level alone or in combination with CA-125. For all EOC cases and for each subtype, the AUC values ranged from 0.48 to 0.72 when using miR-1290 alone, and from 0.83 to 0.97 when using miR-1290 together with CA-125 (Table 2). Particularly, the highest AUC (0.97) obtained using the combination of miR-1290 and CA-125 belonged to the high-grade serous carcinoma subtype. Furthermore, a significantly higher expression of this miRNA in patients with high-grade serous than those with other non-high-grade serous subtypes was also observed. An AUC value of 0.79 was obtained when using a combination of miR-1290 and CA-125 to differentiate high-grade serous from other non-high-grade serous subtypes (Table 2). The authors concluded that miR-1290 can be used as a potential diagnostic biomarker for high-grade serous carcinoma.

The same group also examined the possibility of using exosomal miR-99a-5p, identified using the same method, to predict EOCs [46]. The authors further showed significant up-regulation of circulating cell-free miR-99a-5p in the serum of EOC patients as compared with patients with benign tumor or healthy subjects (Table 1). However, differences in the expression between subtypes were not observed for this miRNA. MiR-99a-5p showed sensitivity of 0.85 and specificity of 0.75 with an AUC of 0.88 for the detection of EOCs. In combination with CA-125, the AUC increased to 0.95. Even though the combination markers failed to distinguish different subtypes, it showed high accuracy in detecting EOC in the early stages (stage I-II) with an AUC of 0.91 (Table 2). 

A recent study by Kim et al. hypothesized that the dysregulation of miRNAs in the EOC tissues should also be observed in the circulating exosomes [47]. The authors, thus, selected seven candidate miRNAs, namely miRNA-21, -93, -141, -145, -200a, -200b and -200c, which are either up-regulated of down-regulated in the EOC tissues (mostly high-grade serous subtypes) from the literature [52,53,54,55]. Using real-time quantitative PCR, they observed significant up-regulation of exosomal miR-21, -93, -145 and -200c in the serum of EOC patients comparing to subjects with benign or borderline tumor (Table 1). Furthermore, up-regulation of miR-21 and -93 was specific in non-high-grade serous subtypes, while high miR-200c expression was specific in high-grade serous carcinoma. Among the four exosomal miRNAs, two demonstrated good EOC-detecting accuracy with miR-145 showing 91.7% sensitivity, 75% specificity and an AUC of 0.910, and miR-200c showing 72.9% sensitivity, 90% specificity and an AUC of 0.802. (Table 2). Different combinations of the two miRNAs with or without CA-125 improved sensitivity but showed lower specificity to detect EOCs (Table 2). Therefore, the author proposed that using exosomal miR-145 alone might be the most promising diagnostic biomarker for EOCs. In addition, they also found that up-regulation of miR-145 and -21 were significantly associated with distant metastasis in high-grade serous carcinoma patients. 

Multiple studies suggested that miR-200c maybe a promising biomarker for detecting EOC, and it is also the most dysregulated miRNA in the circulatory system [56,57]. However, it showed high specificity to high-grade serous carcinoma, but lacked the power to distinguish between different subtypes of EOCs. The other members of miR-200 family including miR-200a and miR-200b, showed incongruent results in different studies. The two miRNAs were observed to be up-regulated in high-grade serous carcinoma in two studies [42,44], but were found to be lowly expressed in all subtypes in another study [47]. The inconsistency may result from different ethnic populations (Caucasians and Asians), or from different sources of exosomes (serum vs. plasma). This raises important concerns for identifying biomarkers used in liquid biopsy; different ethnic populations and different sources of biofluid for biomarker identification should always be taken into consideration when comparing the studies and analyzing the results. 

The candidate exosomal miRNAs reviewed in this article were selected either based on the miRNA profiles in tumors [43,44,47] or by exosomes secreted by cancer cell lines [45,46]. They were not identified by large-scale screening of exosomal miRNAs in patient subjects. It is known that the expression of miRNAs in tumor tissues may not be consistent with the expression of circulating exosomal miRNAs [58,59]. For example, miR-145 has been reported to be significantly down-regulated in EOC tissues, particularly in high-grade serous carcinoma [52,53,54,55]. However, it is significantly up-regulated in the serum exosomes of EOC patients [47]. This indicates that there may be undiscovered selecting and sorting mechanisms, which control the encapsulation of specific miRNAs into exosomes before they are released to the tumor microenvironment for cell-cell communication [59,60,61]. Taken together, the optimal biofluid source, the availability of large case-control cohorts and the independent validation cohorts preferably with a substantial mixture of different ethnic population are required for the successful clinical translation of circulating exosomal miRNA biomarkers. Moreover, the high-throughput exosome isolation technology that can accurately capture circulating exosomes in a faster timeframe would greatly expedite the development of translational applications of exosome.

## 4. Circulating Exosomal miRNA and Cell-Free miRNA Expression Profiles in Different Blood-Based Sources

Plasma and serum are major biofluid sources in biobank repositories worldwide, which provide the most important resources for biomarker identification. As we mentioned in the previous section, the incongruence of reported exosomal miRNA expression may result from the use of different exosome sources (serum vs. plasma). In addition, the differences in the expression profiles of exosomal miRNAs and circulating cell-free miRNAs (cf-miRNA) between different sources have not yet been extensively reviewed. In this section, we focus on the literature that compared the expression patterns of exosomal miRNA and cf-miRNA between serum and plasma. We also discuss two studies that analyze miRNA profiles in the platelets, as there is increasing evidence that platelets are important sources of biomarkers, particularly for the diseases related to platelet dysfunctions, such as cancers [62,63,64]. 

Plasma and serum possess fundamental differences due to their distinct collection processes. Blood tubes for plasma collection contain EDTA to prevent coagulation. In addition, most biobanks collect plasma by centrifugation at high speed for a long period of time, i.e., at 1000–2000× *g* for 10–15 min, to separate cells and biofluid. As a result, the platelets are mixed with buffy coat layer (white blood cell layer) due to the high-speed centrifugation, and we are left with platelet-poor-plasma. For serum collection, blood tubes contain clot-activator, which result in the activation of platelets and the releasing of biological molecules, such as protein, DNA, RNA and microparticles (also known as extracellular vesicles), during the coagulation process [65]. Thus, the slightly different yet important fraction of exosomes related to platelet function may be found in serum, but not in plasma. This fact emphasizes the importance of exosome source selection for biomarker identification.

Hemolysis is one of the important confounding factors in cell-free miRNA biomarker discovery and may also affect the clinical interpretation if the hemolysis-susceptible miRNAs are used as a diagnostic/prognostic marker [66]. Several miRNAs have been used as potential indicators to evaluate hemolysis as they are relatively stable across all sources (e.g., miR-23a), or enriched in red blood cells (RBC) (e.g., miR-144, -16, -451, -486 and -92a) and in white blood cells (WBC) or platelets (e.g., let-7a, miR-150, -197, -199a, -223 and -574) [67,68,69]. Particularly, miR-223 is proposed to be abundantly released by activated platelets [70]. Juzenas et al. (2017) comprehensively analyzed the miRNA expression profiles from seven types of peripheral blood cells, serum, serum exosomes and whole blood [68]. They compared miRNAs in RBC, exosomal miRNAs and cf-miRNAs in serum, and suggested that the miRNAs commonly used as RBC-specific markers can also be found in exosomes or serum. For example, miR-16-5p and -451a apparently could be found across RBC, exosomes and serum, and thus their roles as hemolysis indicator may need to be reconsidered. Other markers such as miR-144-3p were found in RBC and exosomes but was absent in serum, while miR-144-5p could only be found in RBC but not in exosomes and serum. Based on the results of this study, several miRNAs were RBC-specific, including miR-142-3p, -454-3p, -19a-3p, -15b-3p and -421. In addition to using RBC-specific miRNAs to evaluate hemolysis effect, one study has suggested that the ratio between miR-23a and miR-451 can be an indicator of possible RBC lysis, which results in much higher concentration of miR-451 in the plasma and serum [67]. However, it is relatively difficult to define an optimal cutoff value for interpreting the extent of hemolysis. 

One of the first studies published in 2008 by Hunter et al. first examined the expression of miRNAs in exosomes isolated in plasma and matched peripheral blood mononuclear cells (PBMC) from 51 healthy subjects [71]. The expression profiles of a total of 420 miRNAs were examined by reverse transcription quantitative real-time PCR (RT-qPCR). Among those, the authors identified miR-223 to be the most abundant miRNAs in both exosome and PBMC. The results showed that a quantity of miR-223 are more than 10 times higher than the second most abundant miRNA, miR-484, in both sources. The authors further compared the miRNA profiles in platelets isolated from 6 donors and in plasma-derived exosomes. Interestingly, miR-223 is also the most abundant miRNA in platelets, while one of the most abundant miRNAs, miR-484, was not detected in the platelets (Table 3). 

Wang et al. (2012) published a comprehensive study not only compared the amount of cf-miRNAs in different blood-based sources, but also compared them using different probe-based RT-qPCR technologies, i.e., Taqman and LNA (locked nucleic acid) [72]. Even though the study did not analyze miRNA profiles in exosomes, the results are informative and can be a reference for comparing source-specific cf-miRNA and exosomal miRNA profiles. A total of 6 healthy donors were recruited and the cf-miRNA profiles in their plasma, serum, platelets and blood cells (RBC and WBC) were analyzed by either both or only one technique. Overall, the study showed that although the serum and plasma shared cf-miRNA contents, the profiles are different between the two sources (Table 3). Furthermore, the results between the two qPCR platforms showed low consistency (Table 3), probably because of the different pre-amplification steps. In line with other studies, miR-223 is the most abundant cf-miRNAs across various blood-based sources. The authors observed higher RNA concentration in serum than plasma and suggested that RNA/miRNAs may be released from blood cells and platelets into serum during coagulation process. Therefore, plasma may be the sample of choice when studying circulating cf-miRNAs, as RNA released during the coagulation process may change the true cf-miRNA repertoire.

Cheng et al. (2014) aimed to compare the profiles of intracellular miRNAs from peripheral blood cells, and cf-miRNAs and exosomal miRNAs in serum and plasma [73]. Exosomes were further isolated using differential ultracentrifugation (UC) or by commercial exosomal miRNA isolation kits (Norgen Biotek), and miRNAs were analyzed by next generation sequencing (NGS). For plasma, the UC protocol was superior to the commercial exosome isolation column as it did not pellet non-exosomal RNA or cellular RNA contaminants. The commercial kit performed better with serum samples and showed the same RNA profile as those observed in UC-isolated serum exosomes. This finding underscores the importance of standardizing sample collection, centrifugation of blood and handling for exosomal miRNA research. Overall, the results showed that miR-451a and miR-223-3p are the most abundant miRNAs across all samples for both cf-miRNAs and exosomal miRNAs isolated by different methods in plasma and serum. The other abundant miRNAs included miR-191-5p, -486-5p, 484, -16-5p, -126-3p. The authors also compared intracellular miRNAs profiles with those of the cell-free blood and exosomes to identify the unique miRNAs in each group for serum or plasma samples. Interestingly, not only exosomal miRNAs were resistant to RNaseA treatment, but there were also more miRNAs stably present in exosomes compared with cell-free fractions. Hence, the authors concluded that exosomes provide a good protection to miRNAs and therefore appear to be a better source for biomarker identification. Of note, as the authors did not perform exosomal miRNA profile comparison between serum and plasma, we, thus, carried out this analysis based on their results using the same inclusion criteria (miRNAs with mean reads per million (RPM) larger or equals to five). There are 5 and 17 exosomal miRNAs specifically found in plasma and serum, respectively (Table 3). 

Other studies also identified miRNAs commonly seen in serum and plasma by using different platforms [67,74,75,76] including using Nanostring system, which performs quantification by digital counting of RNA molecules without PCR amplifications [75] (Table 3). Unfortunately, these studies only compared cf-miRNAs but not exosomal miRNAs. Of note, one of the studies identified that the abundant RBC-enriched miR-144, 451 and 486 were prevalent in plasma, while the platelet and PBMC-enriched miR-223 and 199a-5p were prevalent in serum [76]. Since biological diversity between patients may contribute to low reproducibility of miRNA profile, this study also investigated the effect of gender, fasting state and menstrual cycle on cf-miRNA levels in serum and plasma and concluded that these factors do not significantly affect cf-miRNA profiles and need not be controlled for [76]. 

In sum, the advantage of using exosomal miRNAs as biomarkers are that they are more stable in archival blood-based biospecimens (i.e., serum and plasma in biobanks) and resistant to ribonuclease degradation. No consensus has yet emerged on which specimen source is best for exosome work. On the contrary, in order to avoid miRNA contamination from blood cells, the specimen of choice for circulating cf-miRNA biomarker discovery is platelet-depleted plasma generated immediately after blood collection.

## 5. Concerns Regarding Endogenous Controls for Exosomal and Cell-Free miRNA Biomarkers

Reference genes/miRNAs have two main purposes: for evaluating techniques of miRNAs isolation as they are usually in a very small amount especially in cell-free blood or in the exosomes (spike-in or exogenous controls), and for normalization of expression levels across samples (endogenous controls). The most commonly used spike-in control is a miRNA from *Caenorhabditis elegans*, cel-mir-39, and it is added into the specimen lysate. The quantity of cel-mir-39 can help identify whether the isolation procedures is successful and consistent between samples. The most commonly used endogenous controls for circulating cf-miRNA are small nuclear and nucleolar RNA, such as U6, RNU44, RNU43 and RNU48 [77]. 

It is worth noting that the literature reviewed above used different reference genes for normalizing expression levels of exosomal and cf-miRNAs, implying that the selection of appropriate reference genes has not yet been standardized (Table 1 and Table 3). It is particularly challenging to identify suitable reference genes for exosomes in different blood-based sources. Several studies identified most stable miRNAs for either cell-free or exosomal miRNAs, which may serve as reference genes in different specimen sources, using various platforms [67,75,78]. However, to use one or a set of miRNAs as universal reference genes for either cell-free and exosomal miRNAs are still under debate. One study using RT-qPCR platform proposed the use of cf-miR-23a as reference as it was relatively stable in plasma and serum and was not affected by hemolysis [67], while the other study using NanoString platform found that cf-miR-30e-5p is the most stable miRNA in both serum and plasma [75]. Gouin et al. applied two different platforms, namely NGS and NanoString miRNA panels, to identify the most stable exosomal miRNAs secreted by cardiosphere-derived cells from healthy donors [78]. Using a combination of four different algorithms (NormFinder, GeNorm, BestKeeper and delta Ct), the authors identified that exosomal miR-23a-3p was present and stably expressed across all samples. They further suggested that a combination of multiple exosomal miRNAs, including miR-23a-3p, miR-101-3p and miR-26a-5p, may yield stronger reference for normalization. Even though multiple studies showed the stability of miR-23a expression in both cf-miRNA and exosomal miRNA fractions, its stability may vary based on different types of tissue or cells that secrete exosomes. In a more recent study, Dai et al. combined RNA sequencing (RNA-Seq) data of exosomes in serum from three different cancer types (pancreatic adenocarcinoma, colorectal carcinoma and hepatocellular carcinoma) and from healthy donors to identify the most stable exosomal miRNAs across samples [79]. The candidate exosomal miRNAs were further verified in serum exosomes of EOC patients. Six exosomal miRNAs were observed to be stably expressed in the discovery cohort (pooled cancers and healthy donors) and validation cohort (EOCs). They are miR-125-5p, miR-192-3p, miR-4468, miR-4469, miR-6731-5p and miR-6835-3p. Among the six exosomal miRNAs, the combination of miR-4468 and miR-6835-3p gave the highest expression stability in both cohorts. It is worth mentioning that two exosomal miRNAs studies in EOC patients chose to use miR-484 over U6 as endogenous controls [43,44] (Table 1). Our preliminary unpublished data also showed that serum exosomal miR-484 has the smallest variation across healthy subjects, patients with benign tumors and patients with EOC. Furthermore, exosomal miR-484 has been used as endogenous control in the serum of breast cancer patients as well [48]. 

Taken together, we conclude that circulating exosomal and cf-miRNA profiles can be affected by many factors. These factors include individual genetic variations, specimen sources, various preanalytical factors including the extent of hemolysis, miRNA isolation protocols, different detection platforms (e.g., RT-qPCR, NanoString or NGS) or different qPCR techniques (e.g., Taqman and LNA assays), and the selection of reference genes. In order to be able to implement exosomal or cf-miRNA biomarkers in clinical setting, these factors will need to be carefully considered and standardized. Thorough evaluations of contamination of miRNAs from disrupted blood cells is suggested before using the data for biomarker identification. Finding a suitable set of standard reference genes for each specific setting remain one of the most challenging tasks for now. 

## 6. Summary of Current Clinical Trials Using miRNAs as Biomarkers in Epithelial Ovarian Cancer

We explore clinical trials registered during years 2016–2021 in ClinicalTrials.gov by using key words “miRNA” or “microRNA” and set the disease to “ovarian cancer”. A total of 12 projects were identified by the keyword search. Among these, only six trials are related to EOC. Two studies led by the same team in China, NCT03738319 and NCT03742856, are recruiting EOC patients for studying circulating exosomal miRNAs and long noncoding RNAs (lncRNA), and multi-omics analysis, respectively (Table 4). For NCT03738319, which focuses on identifying differential expression of exosomal miRNAs and lncRNAs in EOC patients, the team only recruit patients with high-grade serous carcinoma. The first stage aims to recruit 20 patients with high-grade serous carcinoma and 20 participants with benign gynecologic disease for prediction model construction. The second stage aims to recruit 120 participants with suspected high-grade serous carcinoma for validation. For multi-omics study (EOC subtype not specified), the trial plans to conduct whole-exome sequencing, transcriptome sequencing as well as obtaining data from proteomics and metabolomics studies. Of note, the status of these two trials is currently listed as “unknown”. 

The other three clinical trials focus on the drug resistance and treatment response (Table 4). NCT02758652 aims to collect plasma, urine and tumor tissues from EOC patients to elucidate the expression profiles of miRNA. The results will be correlated with treatment responses, progression-free survival (PFS) and overall survival (OS) rate. NCT01391351 aims to search for predictors of therapeutic response, particularly for the combination of Taxol and Carboplatin or the combination of Taxol, carboplatin and avastin. MiRNA expression levels of the enrolled patients will be measured in serum on day 1 of receiving each course of treatment or before surgery. The ovarian cancer arm of NCT03776630 focuses on validating the prognostic value of plasma miR200b with regards to PFS after up-front or post-chemotherapy debulking and adjuvant chemotherapy. The last active clinical trial, NCT03877796, aims to verify a current commercial artificial intelligence (AI) algorithm, Drug Response Predictor (DRP), in EOC by using miRNAs from the formalin-fixed, paraffin-embedded (FFPE) tissue samples of patients to predict their response to investigational cancer drugs (Table 4).

## 7. Conclusions and Future Perspectives

In this review, we focus on the literature that observed significant dysregulation of exosomal miRNAs in EOC and showed the estimation of their sensitivities, specificities and AUC values in detecting EOC or the specific subtypes. We then discuss in detail about the factors that may influence the reproducibility of circulating exosomal and cf-miRNA biomarkers including the selection of biofluid source and normalization reference genes. However, we have noticed that the majority of published articles selected candidate exosomal miRNAs from the literature. The candidate exosomal miRNAs were not identified using a comprehensive screening of large number of miRNAs, which may require high-throughput deep sequencing technology, to acquire the complete catalogue of circulating exosomal miRNAs in EOC patients. A study by Elias et al. [80] is the first to combine NGS analysis of serum circulating cf-miRNA with machine learning techniques, namely a neural network model, to develop a diagnostic algorithm for EOC. This model, which had the AUC value of 0.90, significantly outperformed CA-125 and functioned well regardless of patient age, histology or stage; thus, ushering in the new era of machine-learning-driven biomarker discovery. Multiple studies have used machine learning algorithms to increase the prediction robustness of their miRNA prognostic biomarkers for EOC since then [81,82,83]. So far, the published candidate exosomal miRNA markers are mainly tested in the general EOC patients or only in high-grade serous carcinoma subtype, likely because it has the highest incidence. The other subtypes are gaining more attention lately because some of them show particularly higher prevalence in certain populations, i.e., clear-cell carcinoma has higher incidence in Asians than in Caucasians. Clear-cell carcinoma is also more resistant to chemotherapy, resulting in a higher mortality rate in general. Therefore, a set of exosomal miRNA biomarkers showing high sensitivity and specificity for every subtype would be highly beneficial.

Future work to accelerate the clinical application of exosomal miRNAs markers for early detection of EOCs may include: (1) using a larger cohort with adequate numbers of subjects for each subtype to provide a more powerful prediction accuracy; (2) recruiting subjects from different ethnic groups as EOC subtypes show various incidences in different populations; (3) the development of consensus protocols for biofluid (serum and plasma) collection, processing and long-term storage; (4) using the standardized high-throughput exosomal miRNA isolation, characterization and profiling platforms to better understand the biology underlying exosomal miRNAs; and (5) using multiple machine learning algorithms to identify candidates in different subtypes. With these solutions, we hope a prediction model for circulating exosomal miRNAs that can accurately diagnose EOC at an early stage can be realized in the near future.

## Figures and Tables

**Table 1:** Circulating exosomal miRNAs identified as potential diagnostic biomarkers in EOCs. N/A: not available, HGSC: high-grade serous carcinoma.

References	Exosomal miRNAs	Bioliquid	Subtypes	Expression Pattern	Normalization Controls	Detection Methods
Taylor and Gercel-Taylor 2008 [42]	miR-21, miR-200a/b/c, miR-141, miR-203, miR-205, miR-214	Serum	HGSC	Up-regulated in HGSC	N/A	miRNA array
Meng et al., 2016 [43]	miR-200a/b/c miR-373	Serum	EOCs	Up-regulated in EOCs	miR-484 cel-miR-39	Taqman assay
Pan et al., 2018 [44]	miR-21 miR-100 miR-200b miR-320	Plasma	EOCs	Up-regulated in EOCs	RNU6, miR-484, cel-miR-39	Taqman assay
	miR-16 miR-93 miR-126 miR-223	Plasma	EOCs	Down-regulated in EOCs		
Kobayashi et al., 2018 [45]	miR-1290*	Serum	HGSC	Up-regulated in HGSC Down-regulated in non-HGSC	cel-miR-39	Taqman assay
Yoshimura et al., 2018 [46]	miR-99a-5p*	Serum	EOCs	Up-regulated in EOCs	N/A	Taqman assay
Kim et at., 2019 [47]	miR-145	Serum	EOCs	Up-regulated in EOCs	RNU48	Taqman assay
	miR-200c	Serum	HGSC	Up-regulated in HGSC		
	miR-21 and miR-93	Serum	non-HGSC	Up-regulated in non-HGSC		

* These exosomal miRNAs were first identified in HGSC cell lines. Circulating cell-free miRNAs were then quantified in the patient sera.

**Table 2:** Sensitivity, specificity and area under the receiver operating characteristic (ROC) curve (AUC) of the circulating exosomal miRNAs used to detect epithelial ovarian cancers (EOCs) in the literature. HGSC: high-grade serous carcinoma, CCC: clear-cell carcinoma, ENC: endometroid carcinoma, MUC: mucinous carcinoma.

References	Exosomal miRNAs	Detected Subtype *	Sensitivity	Specificity	AUC	No. of Subjects
Meng et al., 2016 [43]	miR-200a	EOCs	0.839	0.900	0.914	HGSC *n* = 120; non-HGSC *n* = 15; unknown subtype *n* = 28; benign tumor *n* = 20; healthy *n* = 32
	miR-200b	EOCs	0.528	1.000	0.815	
	miR-200c	EOCs	0.311	1.000	0.655	
	miR-200a + b + c	EOCs	0.882	0.900	0.925	
Pan et al., 2018 [44]	miR-21	EOCs	0.610	0.820	0.740	HGSC *n* = 90; non-HGSC *n* = 13; unknown subtype *n* = 3; ovarian cystadenoma *n* = 8; healthy *n* = 29
	miR-100	EOCs	0.620	0.730	0.710	
	miR-200b	EOCs	0.640	0.860	0.868	
	miR-320	EOCs	0.560	0.690	0.658	
Kobayashi et al., 2018 ** [45]	miR-1290	EOCs	0.510	0.570	0.480	HGSC (*n* = 30); CCC (*n* = 18); ENC (*n* = 12); MUC (*n* = 10)
	miR-1290 + CA-125 ^a^	EOCs	-	-	0.920	
	miR-1290	HGSC	0.630	0.850	0.710	
	miR-1290 + CA-125	HGSC	-	-	0.970	
	miR-1290	CCC	0.580	0.890	0.690	
	miR-1290 + CA-125	CCC	-	-	0.940	
	miR-1290	ENC	0.500	0.830	0.620	
	miR-1290 + CA-125	ENC	-	-	0.910	
	miR-1290	MUC	0.580	0.900	0.720	
	miR-1290 + CA-125	MUC	-	-	0.830	
	miR-1290	HGSC vs. non-HGSC	0.470	0.850	0.760	
	miR-1290 + CA-125 ^b^	HGSC vs. non-HGSC			0.790	
Yoshimura et al., 2018 ** [46]	miR-99a-5p	EOCs	0.850	0.750	0.880	HGSC *n* = 32; CCC *n* = 15; ENC *n* = 9; MUC *n* = 6; Early stage (stage I-II) EOCs *n* = 31; benign tumor *n* = 26; healthy *n* = 20
	miR-99a-5p + CA-125 ^c^	EOCs	-	-	0.950	
	miR-99a-5p	Early stage EOCs	0.900	0.750	0.850	
	miR-99a-5p + CA-125 ^d^	Early stage EOCs	-	-	0.910	
	miR-99a-5p	EOCs vs. benign tumor	0.870	0.540	0.700	
	miR-99a-5p + CA-125 ^e^	EOCs vs. benign tumor	-	-	0.810	
Kim et al., 2019 [47]	miR-21	EOCs	-	-	0.585	HGSC *n* = 39; non-HGSC *n* = 9, borderline tumor *n* = 10; benign ovarian cyst *n* = 10
	miR-93	EOCs	-	-	0.755	
	miR-145	EOCs	0.917	0.750	0.910	
	miR-145 + CA-125 ^f^	EOCs	0.979	0.600	-	
	miR-200c	EOCs	0.729	0.900	0.802	
	miR-200c + CA-125 ^f^	EOCs	0.938	0.700	-	
	miR-145 + miR-200c	EOCs	0.938	0.650	-	
	miR-145 + miR-200c + CA-125 ^f^	EOCs	1.000	0.550	-	

* The detected group as compared with healthy controls (unless stated otherwise). ** These two studies first identified exosomal miRNAs in cell lines and then investigated the circulating cell-free miRNAs in the patient sera. ^a^ CA-125 alone had an AUC of 0.900; ^b^ CA-125 alone had an AUC of 0.690; ^c^ CA-125 alone had an AUC of 0.910; ^d^ CA-125 alone had an AUC of 0.840; ^e^ CA-125 alone had an AUC of 0.790; ^f^ CA-125 alone had an AUC of 0.801.

**Table 3:** Exosomal miRNAs and circulating cell-free miRNAs showing differential expression between various bioliquid sources. N/A: not available.

Reference	Analytes	miRNA Sources	miRNAs	Detection Techniques	Normalization Controls	Contamination Indicators
Hunter et al., 2008 [71]	Exosomal miRNAs	Plasma,	**Higher in exosomes**: miR-486, 328, 183, 32, 574, 27b, 222, 197, 151, 199a, 133b, 320, 96, 103, 17-5p	RT-qPCR panel (420 miRNAs)	RNU38B, RNU43, U6, 5S and 18S rRNA	N/A
		PBMC	**Higher in PBMC**: miR-150, 29a, 142-3p, 146b, 155, 532, 19a, 140, 21, 374, 181d, 345, let-7g, 15a, 19b, 142-5p, 106b, 26b, 195			
		Platelets	**Most abundant miRNA**: miR-223 (**Note**: miR-484 is not detected)			
Wang et al., 2012 [72]	Circulating cell-free miRNAs	Plasma	**Most abundant in plasma**: Taqman: miR-126, 146a, 150, 19b, 222, 223, 451, 617, 92a Exiqon: miR-15a, 16, 19b, 1974, 21, 223, 451, 486-5p, 92a	RT-qPCR (Taqman and Exiqon)	U6, RNU44 and RNU48	miR-150 as WBC lysis indicator; miR-16 as RBC lysis indicator; miR-126 as platelet activation indicator
		Serum	**Most abundant in serum**: Taqman: miR-17, 146a, 19b, 223, 24, 451, 519c, 92a Exiqon: miR-16, 126, 142-3p, 19b, 1974, 223, 451, 92a, 486-5p, 720			
		Platelets	**Most abundant in platelet**: Exiqon: miR-126, 16, 142-3p, 19b, 21, 223, 451			
Cheng et al., 2014 [73]	Exosomal miRNAs	Plasma and serum	**Most abundant in both plasma and serum**: miR-126-3p, 16-5p, 191-5p, 223-3p, 451a, 484, 486-5p	Next-generation sequencing (NGS)	Reads per million mapped reads (RPM)	N/A
		Plasma	**Specific in plasma**^a^: miR-664a-5p, 654-5p, 3620-3p, 4446-3p, 877-5p			
		Serum	**Specific in serum**^a^: miR-196b-5p, 502-3p, 16-2-3p, 550a-5p, 1180, -7-5p, 4732-5p, 532-3p, 204-5p, 942, 183-5p, 629-5p, 214-3p, 1292-5p, 550a-3p, 550b-2-5p, 500a-3p			
Blondal et al., 2013 [67]	Circulating cell-free miRNAs	Plasma and serum	119 miRNAs are found in both plasma and serum. Please see Appendix A in Blondal et al., 2013.	RT-qPCR (Exiqon)	Exiqon RNA spike-in kit	miR-23a-3p: stability indicator; miR-451: RBC lysis indicator
Ammerlaan and Betsou 2016 [74]	Circulating cell-free miRNAs	Plasma	**Stably expressed in plasma**: Let-7e*, miR-100, 105*, 106b*, 1228*, 1288, 1469, 150*, 1538, 183, 19b-1*, 3193, 320c, 342-3p, 342-5p, 3652, 3918, 3937, 4325, 503, 664, 92b*, 939, 99a	SmartChip Human miRNA Panel V3.0 (WaferGen)	Spike-in kit (WaferGen)	N/A
		Serum	**Stably expressed in serum**: Let-7d*, miR-106b*, 1231, 1237, 1273c, 1285, 1294, 1306, 142-5p, 155, 222, 29b, 302d*, 31, 3180-3p, 3192, 3652, 370, 371-5p, 423-3p, 4252, 4278, 4286, 4297, 503, 543, 611, 675, 873, 99a			
Foye et al., 2017 [75]	Circulating cell-free miRNAs	Plasma and serum	**Most abundant in both serum and plasma**: miR-128-1-5p, 19b-3p, 26a-5p, 302a-5p, 543, 544, 548g-3p, 585-3p, 6721-5p	NanoString Human miRNA panel	Background subtraction and total mean normalization	miR-24-5p as WBC lysis indicator; miR-16-5p and miR-15b-3p as RBC lysis indicator ^b^
			**Most dysregulated between serum and plasma**: Let-7b-5p, miR-126-3p, 144-3p, 16-5p, 191-5p, 223-3p, 25-3p, 451a, 4454 + 7975, 873-3p			
Max et al., 2018 [76]	Circulating cell-free miRNAs	Plasma	**Top 10 up-regulated in plasma**: miR-144, 16-2 *, 18b, 3158, 3200, 451, 4685, 486, 517a, 550-1-3p	NGS	DESeq2 normalization	RBC-enriched miR-144, 451 and 486 are higher in plasma, and the platelet and PBMC-enriched miR-223 and 199a-5p are higher in serum.
		Serum	**Top 10 up-regulated in serum**: miR-223, 2355-5p, 411, 432, 487b, 493-5p, 495, 543, 582, 889			

* Indicates the opposite arm of the predominant miRNAs. ^a^ Results from further analysis by the authors of this review article. ^b^ Foye et al. identified higher WBC contamination in plasma.

**Table 4:** Summary of current clinical trials related to epithelial ovarian cancer that use miRNAs in the circulatory system or tissues as biomarkers. FIGO: Federation of Gynecology and Obstetrics.

NCT Number *	Study Title	EOC Subtype	Specimen	Expected Outcome **
NCT02758652	Molecular Mechanisms Leading to Chemoresistance in Epithelial Ovarian Cancer (CHEMOVA)	Not specified	Plasma	miRNA expression profiles of ovarian cancer patients in 5 years of trial period
NCT03776630	Exploring the Potential of Novel Biomarkers Based on Plasma microRNAs for a Better Management of Pelvic Gynecologic Tumors (GYNO-MIR)	Not specified	Plasma	To validate the previous finding on the prognostic value of the pre-/post-treatment variation of miR-200b concentrations in plasma with regards to progression-free survival (PFS)
NCT01391351	Search for predictors of therapeutic response in ovarian carcinoma	Not specified	Serum	Identify predictors of response to chemotherapy in ovarian carcinoma patients using the miRNA profile in serum before chemotherapy treatment
NCT03738319	Non-coding RNA in the exosomes of epithelial ovarian cancer	High-grade serous	Blood	Identify miRNA and lncRNA in exosomes of high-grade serous carcinoma as detection and prognostic biomarkers
NCT03742856	A Multi-omics Study of Epithelial Ovarian Cancer	Not specified	Blood and cancer tissue	The alteration of RNA expression, including mRNA, miRNA and lncRNA, will be compared between patients of different FIGO stages and different pathological subtypes
NCT03877796	Clinical Pre-screening Protocol for Ovarian Cancer	Not specified	FFPE tissue block	Identification of ovarian cancer patients with high likelihood of being sensitive to investigational cancer drug based on FFPE ovarian cancer tissue (Drug Response Predictor^®^ (DRP))

* Registered data between 2016–2021 was last extracted from https://clinicaltrials.gov/, accessed on 3 July 2021. ** Only outcomes related to the use of miRNA expression as biomarkers are listed.

## Data Availability

All the articles cited in this review can be found in Pubmed (https://pubmed.ncbi.nlm.nih.gov/ accessed on 3 July 2021).
